# The metastatic niche and stromal progression

**DOI:** 10.1007/s10555-012-9373-9

**Published:** 2012-06-15

**Authors:** Jonathan P. Sleeman

**Affiliations:** 1 Centre for Biomedicine and Medical Technology Mannheim (CBTM), Universitätsmedizin Mannheim, University of Heidelberg, TRIDOMUS-Gebäude Haus C, Ludolf-Krehl-Str. 13-17, 68167 Mannheim, Germany; 2 KIT Karlsruhe Campus Nord, Hermann-von-Helmholtz-Platz 1, 76344 Eggenstein-Leopoldshafen, Germany

**Keywords:** Metastatic niche, Pre-metastatic niche, Microenvironment, Stromal progression, Metastasis, Dormancy

## Abstract

The tumor stroma is comprised of extracellular matrix, non-malignant cells, and the signaling molecules they produce. It is an integral and vital component of primary tumors that together with the underlying genetic defects in the tumor cells determines the growth characteristics, morphology, and invasiveness of the tumor. In parallel to continuing genetic changes in the tumor cells themselves, the tumor stroma progressively evolves during primary tumor development. Cancer cells that disseminate from primary tumors are dependent on this stromal microenvironment, and therefore the microenvironment they encounter at secondary sites determines their fate. For those cells that survive at these sites, stromal progression can serve to re-establish a supportive tumor stroma, fostering the outgrowth of the cells as metastases. Formation of a metastatic niche that supports the survival and growth of disseminated tumor cells is a key feature of this stromal progression. The endogenous organ microenvironment can provide components of the metastatic niche. In addition, microenvironmental changes in organs prior to receipt of disseminated tumor cells can be induced by factors secreted systemically by primary tumors, causing the formation of pre-metastatic niches. Further maturation of metastatic niches can be responsible for the re-activation of dormant disseminated tumor cells many years after removal of the primary tumor. The concept of the metastatic niche and stromal progression has profound consequences for our understanding of metastatic disease, and promises to open up new strategies for the diagnosis, prognostic evaluation, and therapy of cancer.

## Introduction

After invading out of the primary tumor and entering the vasculature, tumor cells can in principle be transported throughout the body. A key step in the formation of metastases is the exit of these circulating tumor cells (CTCs) from the circulatory system and their entry into secondary sites to become disseminated tumor cells (DTCs). In addition to the intrinsic properties of these cells, the microenvironment local to DTCs is critical in determining their fate [1]. The term metastatic niche [2] has been coined to describe the microenvironmental conditions required for the survival and outgrowth of DTCs at these secondary sites. Metastatic niches can be derived from the foundation of particular microenvironments found endogenously in organs where metastases form. Their formation can also be remotely induced at least in part by primary tumors before the arrival and establishment of DTCs (termed pre-metastatic niches). Further remodeling may be required once DTCs have become established so that the development of a fully mature metastatic niche ensues.

The development and evolution of metastatic niches should be seen in the broader context of tumorigenesis and tumor progression as a whole, as suggested by the stromal progression model of metastasis [3]. In this model, the formation of primary tumors is dependent not only on progressive genetic changes in cancer cells but also on the progressive development of an inflammatory tumor stroma. These two processes are mutually dependent on each other in a continual co-evolution. The dependency of primary tumor cells on their stroma means that when they disseminate, the absence of the requisite stroma at secondary sites determines that most if not all of the cancer cells are unable to survive or simply remain dormant, depending on the microenvironment they encounter. The formation of metastatic niches either pre-metastatically or after the settlement of DTCs serves to re-establish the stromal environment that DTCs require for growth as tumors, and can be coupled to further genetic changes in the tumor cells themselves. Thus, the evolution of metastatic niches recapitulates stromal progression in the primary tumor [3].

The concept of the metastatic niche and stromal progression extends Paget’s seed and soil hypothesis [4] through focusing on the properties of the microenvironmental soil needed for successful metastasis formation by the metastatic seed. In recent years, much progress has been made in understanding key components of the metastatic niche, how such niches form and are regulated, and the effects they exert on the tumor cells they interact with. In this review, I survey what we currently know about the form, regulation and function of metastatic niches, and discuss some of the potential clinical implications of their existence.

## Key components of the niche

**Fig. 1:**
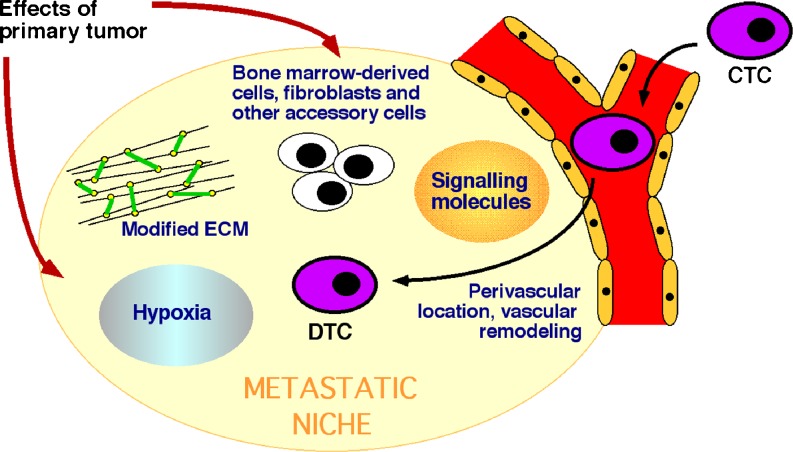
Diagram showing the relationship between CTCs, DTCs, and the metastatic niche. The current concept of the metastatic niche and important components within it are illustrated

Although investigation of the metastatic niche is still a relatively young research field, a number of central functional elements have emerged. These include a perivascular location, modification of the extracellular matrix (ECM) and remodeling of the vasculature, recruitment of bone marrow-derived cells (BMDCs), hypoxia, and the expression of a variety of signaling molecules (Fig. 1). Other non-transformed cells such as endothelial cells and fibroblasts are also important players.

### Modified ECM

Formation of the metastatic niche is associated with the deposition of new ECM components as well as with the remodeling of the ECM constituents. For example, the deposition of fibronectin, tenascin-C, periostin, and versican have all been implicated in metastatic niche formation [5–8]. Accumulation of fibronectin produced by fibroblasts and fibroblast-like cells has been reported to determine at which sites metastatic niches form [5], although at least some of the deposited fibronectin is derived from the primary tumor [9]. Recent studies suggest that this deposition results in coordinated remodeling of the ECM, as periostin acts as a bridge that binds to tenascin-C as well as to fibronectin and collagen type I, thereby serving to promote incorporation of tenascin-C into the ECM [10]. Conversely, reduced levels of the ECM protein fibulin-5 have been reported to be required for metastasis formation in the liver and lung, probably because fibulin-5 suppresses expression of matrix metalloproteinase (MMP)-9 [11], a protease that remodels the ECM during metastatic niche formation and promotes metastatic outgrowth [5, 12]. MMP-2 also plays an important role in organizing the ECM of the metastatic niche [9]. Another class of enzymes, the lysyl oxidases (LOX) and LOX-like proteins (LOXL), are additionally actively involved in ECM remodeling during niche formation due to their ability to cross-link collagen and elastin, and are upregulated in response to hypoxia via HIF-1α [9, 13, 14]. LOX produced in response to hypoxia by primary tumors localizes to sites of fibronectin deposition [9]. In addition to changing the constitution of the ECM and thereby providing substrates for tumor cells and the cellular components of the metastatic niche, an important consequence of ECM remodeling is to increase matrix stiffness, which can profoundly affect the properties of tumors cells [15]. For example, metastasis formation is promoted by Caveolin1 expression on fibroblasts, which serves to remodel and stiffen the ECM microenvironment [16].

### Perivascular location and vascular remodeling

Recent long-term intravital video microscopy experiments using the mouse cranial window model have revealed that, once extravasated, tumor cells require a strict perivascular location if they are to survive [17]. This was true regardless of whether the cells remained as single dormant cells or grew out as metastases after vessel cooption or the induction of angiogenesis. VEGF-A inhibition was observed to push perivascular tumor cells into a state of dormancy [17]. Further work is required to determine whether a perivascular location is strictly required in all secondary organs. Earlier intravital studies in the lung suggested that B16 melanoma cells are randomly distributed 4 days after intravenous injection, but after 10 days are located around arterial and venous vessels [18].

Recent evidence also points to vascular remodeling as an important event during formation of the metastatic niche [19]. Other studies indicate that angiopoietin 2 (Angpt2), MMP-3, and MMP-10 combine synergistically to increase vascular permeability in the lung and disrupt vascular integrity [20]. Disruption of vascular integrity is connected with morphological changes in the endothelial cells and the associated basement membrane, as well as with primary tumor-induced fibrin deposition in the lung [20].

### Cellular niche components

Several types of BMDC are important constituents of the metastatic niche. Hematopoietic progenitor cells that express vascular endothelial growth factor receptor-1 (VEGFR-1) are mobilized and recruited early during metastatic niche formation. These cells express α4β1 integrin (VLA-4), a surface receptor for fibronectin, which mediates their recruitment to fibronectin-rich microenvironments where pre-metastatic niches form [5]. They contribute to ECM remodeling through secretion of MMP-9 [5, 12]. They also contribute to the niche vasculature, both directly [21], and through the generation of a microenvironment that recruits VEGFR2+ circulating endothelial progenitor cells [5]. CD11b+ (Mac-1+) cells are also recruited to metastatic niches [22]. LOX activity at these sites cross-links collagen IV, providing a substrate to which the CD11b+ cells adhere and remodel [9]. Production of MMP-2 by the CD11b+ cells cleaves collagen IV, releasing chemoattractive collagen IV peptides. Together, these events attract further CD11b+ and c-Kit+ BMDC, and recruit metastatic cells [9].

Chronic inflammation is associated with metastasis formation [23–26], and accordingly a variety of inflammatory cells that drive primary tumor growth are important components of the metastatic niche. In addition to CD11b+ myeloid cells, other examples include tumor-associated macrophages (TAMs), CD4+ T-reg cells, and certain classes of neutrophils [27]. Surprisingly little is known about the recruitment of some of these types of immune cell to developing metastases and their contribution to the metastatic niche. CD4+ T-reg cells accumulate in lymph node metastases [28–31]. They are also recruited to and required for lung metastases in experimental breast tumors [32, 33].

Fibroblasts play an important role in metastatic niches [2]. They produce a variety of growth factors and cytokines such as S100A4, TGFβ, and SDF1a [34]. As pointed out above, they constitute an important source of fibronectin [5]. They also produce tenascin-C, which augments that produced by breast cancer cells in micrometastatic lesions [6]. The importance of this stromally derived tenascin-C is demonstrated by absence of lung metastasis formation when breast cancer cells are implanted into tenascin-C-deficient mice [35]. Furthermore, periostin is produced by stromal fibroblasts in response to TGFβ3 [7]. Pulmonary fibrocytes contribute to matrix remodeling through expressing MMP-9 [36].

In addition to providing a blood supply for the metastatic niche and contributing to a perivascular microenvironment, endothelial cells also play other roles in the niche. They are a source of the pro-inflammatory cytokines S100A8 and S100A9 that recruit CD11b+ myeloid cells to the niche, and secrete other factors that stimulate the migration of tumor cells [22]. Consistently, PECAM-1 expression on endothelial cells has been reported to stimulate outgrowth of metastases in the lung through a paracrine mechanism [37].

### Hypoxia

Hypoxia in the primary tumor plays an important role in inducing the expression of factors that initiate and regulate formation of metastatic niches, for example VEGF-A, PlGF, and LOX [5, 9, 13, 14]. However, it is also likely that hypoxia within metastatic niches may play an important role in the metastasis-promoting function of the niches. Hypoxia promotes the formation of an inflammatory milieu [38], which supports metastatic outgrowth, and which is likely to an important end point of metastatic niche formation. Moreover, hypoxic microenvironments upregulate SDF1α, and also recruit VEGFR1+ and CD11b+ BMDCs [39].

### Signaling molecules

Growth factors, cytokines, chemokines, and other proteins produced by cellular components of the metastatic niche are pivotal in the formation of metastatic niches, for the attraction of CTCs, and for the survival and outgrowth of DTCs [5, 9, 22, 40]. Members of the Serum Amyloid A (SAA) acute phase proteins whose expression is regulated by pro-inflammatory members of the S100 family such as S100A8 and S100A9 play a central role in the formation of metastatic niches and their function [27]. Monocyte and macrophage-specific chemokines are also constituents of the signaling molecules found in metastatic niches [22].

## Regulation of niche formation

Our understanding of the regulation of metastatic niche formation is still fairly rudimentary. When DTCs arrive at distant sites, the endogenous organ microenvironment may provide some or possibly even all of the components of the metastatic niche structures the DTCs require to survive and grow. The hematopoietic stem cell niche in the bone has been shown, for example, to provide prostate cancer cells with the microenvironment they require for metastasis formation [41, 42]. However, most studies suggest that remodeling of the microenvironment is required to produce a fully competent metastatic niche, either before the arrival of tumor cells and/or after the establishment of DTCs at secondary sites. Research to date has largely focused on primary tumor-induced pre-metastatic remodeling in the lung. The prevalence of endogenous niche structures in particular organs, or organ-specific metastatic niche formation, may underlie patterns of metastatic outgrowth [5]. For example, it is conceivable that the preferential outgrowth of metastases in organs such as lymph nodes, lungs, liver, brain, and bone is due to the presence of endogenous microenvironments in these organs that already contain many metastatic niche components, and can therefore be more readily remodeled to produce metastatic niches. Alternatively or in addition, such endogenous microenvironments may be present at higher densities in metastasis-prone organs than in other organs. Nevertheless, the inefficiency of metastasis formation observed in experimental animals (e.g., [18]) probably suggests that even the most conducive endogenous niche microenvironments are sparsely distributed in organs where metastases develop.

Experimental animal models in which cultured cancer cells are implanted *in vivo* and give rise to metastatic primary tumors have been instrumental in defining how primary tumors can prime future sites of metastasis formation by establishing pre-metastatic niches. A key finding that has emerged from these studies is that factors derived from primary tumors can act in a variety of ways to induce pre-metastatic niche formation. A number of growth factors and cytokines produced by tumors have been shown to have systemic effects that result, for example, in the mobilization and recruitment of BMDC, and ECM remodeling. VEGF-A and PlGF produced by primary tumors mobilize and recruit VEGFR1+ VLA-4+ BMDC to fibronectin-rich pre-metastatic sites in the lung [5]. Factors produced by the primary tumor such as VEGF-A, TNFα, and TGFβ also induce expression of the pro-inflammatory cytokines S100A8 and S100A9 in developing pre-metastatic niches in the lung. In turn, S100A8 and S100A9 induce expression of SAA proteins that then recruit CD11b+ myeloid cells to these sites [22, 40].

Systemic levels of osteopontin produced by primary tumors have also been shown to play a role in activating BMDC [43]. Recent studies further implicate primary tumor-derived coagulants in the recruitment of CD11b+ cells to pre-metastatic niches [44]. As yet undefined primary tumor-derived factors upregulate expression of Angpt2, MMP-3, and MMP-10 in the lung mesenchyme, resulting in disruption of vascular integrity that is required for recruitment of CD11b+ cells [20]. Pre-metastatic induction by primary tumors of lymphangiogenesis in regional lymph nodes is also induced by tumor-produced VEGF-A and VEGF-C [45–47], and is associated with poor prognosis [48]. Our own studies show that by inhibiting these pre-metastatic changes, the outgrowth of metastases in the lymph nodes is inhibited (Quagiata et al., manuscript submitted), providing further evidence of the importance of vascular remodeling in the metastatic niche. Finally, microvesicles and exosomes derived from primary tumors have also been demonstrated to play a role in mediating pre-metastatic changes in putative sites of metastasis formation, for example by inducing expression of VEGF-A, MMP-2, and MMP-9 at these sites [49–52].

The recruitment of BMDC to pre-metastatic sites induces further remodeling of the microenvironment. CD11b+ myeloid cells contribute to modification of the ECM by depositing versican in developing metastatic niches [8]. They additionally secrete large amounts of MMP-9, which remodels the ECM and the vasculature [19, 22, 40]. The recruited VEGFR1+ BMDCs also contribute to MMP-9 expression, together with local endothelial cells [5]. Both VEGFR1+ and CD11b+ cells can incorporate into tumor endothelium and contribute to vascular remodeling, angiogenesis, and vasculogenesis [5, 53, 54]. Loss of systemically acting primary tumor-produced angiogenesis inhibitors upon surgical removal of the primary tumor can also stimulate the outgrowth of metastases [55].

The power of the animal models used to study pre-metastatic niche formation lies in the rapid tumor growth and ease of experimental manipulation. However, they do not faithfully mimic the growth and development of autochthonous tumors that occur in human cancer patients. It therefore remains to be demonstrated the degree to which the development of pre-metastatic niches in human cancer patients contributes to metastasis formation. There are, however, already some indirect indications that this might be the case. For example, for many types of cancer, it is well established that the size of the primary tumor correlates with the probability of metastasis formation [56] (although this is not invariably the case [57]). As we have seen above, soluble factors produced by primary tumors have been identified as initiators of pre-metastatic niche formation. The circulating levels of pre-metastatic niche-inducing factors would be expected to rise as a function of tumor size because the larger the primary tumor, the more of these factors would be expected to be produced. Accordingly, the circulating levels of VEGF and PlGF, initiators of the mobilization and recruitment of VEGFR1+ BMDC, can correlate with tumor size [58–61]. Furthermore, the numbers of circulating VEGFR1+ monocytic cells are reduced in patients with recurrent glioblastoma treated with aflibercept, a recombinant decoy receptor that sequesters VEGF and PlGF [62]. Moreover, clusters of VEGFR1+ BMDCs are observed in putative sites of metastasis formation in breast cancer patients before tumor spread [5].

Pre-metastatic formation of niche structures does not explain why metastases grow out many years after apparent successful therapeutic eradication of the original primary tumor. Viable DTCs can be found in cancer patients many years after surgery without any signs of overt metastases being present [63], and thus are thought to be in a state of dormancy as a consequence of cell cycle arrest [64]. Tumor cells also remain dormant as small clusters of cells known as micrometastases in which there is a balance between proliferation and apoptotic cell death [1]. Genetic changes in the dormant cells, for example loss of metastasis suppressor genes [65] or upregulation of VCAM-1 [66], can stimulate the awakening of dormant tumor cells. However, as discussed below, the induction of metastatic niche formation in the locality of dormant cells may also re-activate them and support subsequent metastatic outgrowth [3]. Our understanding of the induction or maturation of metastatic niches in this context is still at an elementary stage. Pathological events such as chronic inflammation or extensive tissue trauma could conceivably play an initiating role through inducing sustained systemic levels of the growth factors and cytokines that induce metastatic niche formation.

**Fig. 2:**
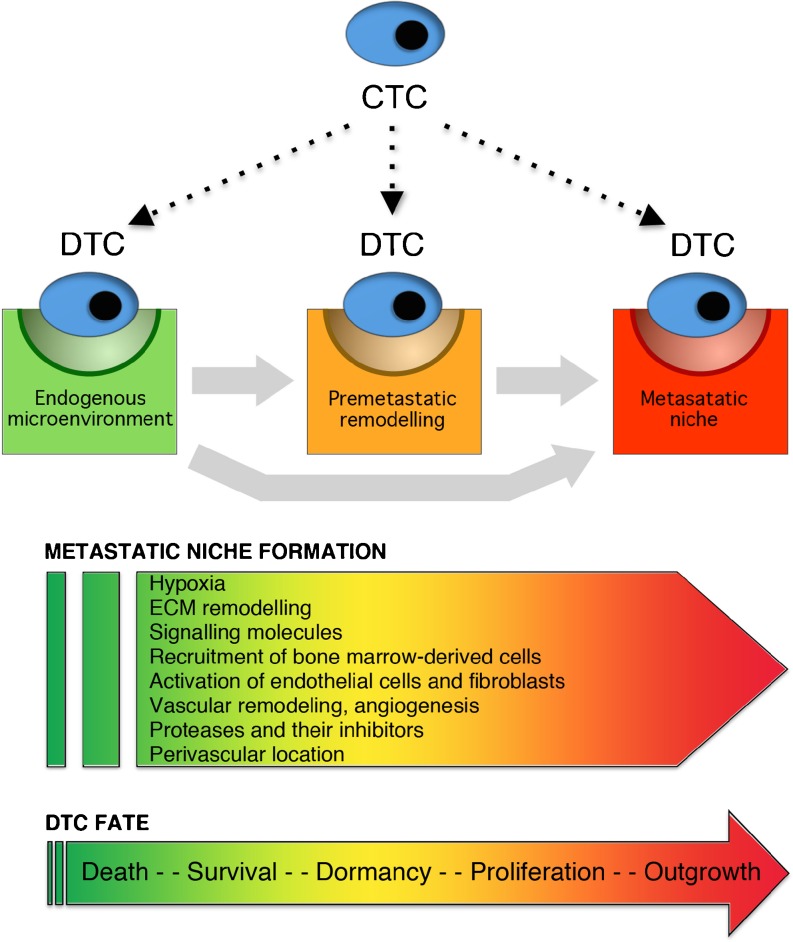
Diagram illustrating our current understanding of stromal progression at sites of metastasis formation. Intrinsic properties of CTCs determine their ability to extravasate into distant organs. In these organs, CTCs encounter either the endogenous niche environment of the organ concerned or a remodeled pre-metastatic niche microenvironment that has been induced by the primary tumor. This pre-metastatic remodeling may be sufficient by itself to produce a metastatic niche that is competent to support metastasis. Alternatively, additional microenvironmental remodeling may be required once a DTC has become established *in situ* to convert either the endogenous organ microenvironment or a partially remodeled pre-metastatic niche into to a fully competent outgrowth-supporting metastatic niche. A number of factors are listed that may be required for the remodeling that results in metastatic niche formation. Various fates are possible for DTCs. These are determined by the intrinsic properties of the DTC as well as by the particular microenvironment that the DTC finds itself in

The picture that emerges from the experimental evidence to date is that starting from the endogenous organ microenvironment, a continual stromal evolution ultimately gives rise to fully mature metastatic niches capable of supporting metastatic outgrowth (Fig. 2). Some of this remodeling can occur pre-metastatically in response to systemic primary tumor-derived factors. Further remodeling can occur once tumor cells occupy nascent metastatic niches. Formation or maturation of metastatic niches can also take place many years after seeding of DTCs. Thus, the development of metastatic niches forms part of a mutually dependent co-evolution of tumor cells and their microenvironment at primary and secondary sites [3].

## Functions of the metastatic niche

The overarching function of the metastatic niche is to determine the fate of DTCs, namely whether they survive, become dormant, or progressively grow to form fulminant metastases. A number of mechanisms have been uncovered that allow them to do this, as outlined below. There are also indications that metastatic niches can signal to and attract CTCs. Accordingly, the different components of the metastatic niche combine to mediate these different functions. Nevertheless, tumor intrinsic properties are also required if tumor cells are to interact productively with the metastatic niche. For example, the metastasis suppressor gene KAI1/CD82 downregulates fibronectin expression and β1 integrin activation in prostate cancer cells [67], thus inhibiting the ability of tumor cells to interact with niche components.

### Attraction of CTCs and stimulation of extravasation

Several components of metastatic niches have been reported to actively induce CTCs to home to the niche. SAA3 produced in response to S100A8 in pre-metastatic lung niches not only recruits CD11b+ cells but also attracts and recruits CTCs to these sites [40]. The VEGFR1+ BMDC and associated fibroblasts produce SDF-1, which recruits CXCR4-positive tumor cells to pre-metastatic niches [5]. Pre-metastatic destabilization of the vasculature in the lung serves to promote the extravasation of CTCs, thereby stimulating the formation of lung metastases [20]. The ECM constitution and conformation in the metastatic niche provides substrates with which integrins and other receptors on the surface of tumor cells can interact (e.g., [5]).

### Establishment of an inflammatory milieu

Arguably one of the most important functions of metastatic niche formation is to foster the formation of an inflammatory microenvironment that recapitulates the tumor–stroma interactions that drive primary tumor growth, and thereby supports metastatic outgrowth. A number of mechanisms in the metastatic niche have been uncovered that act in this way. The induction of S100A8/A9 expression in the pre-metastatic niche that in turn induces expression of SAA family members has emerged as an important inducer of an inflammatory milieu [27]. The S100A8 induced in the pre-metastatic lung in response to factors secreted by primary tumors promotes the expression and secretion of SAA3. In turn, SAA3 recruits myeloid cells and also positively auto-regulates itself via the TLR-4 receptor [40]. TLR-4-expressing Clara cells in the terminal bronchioles participate in this auto-amplification of SAA3 expression. Thus, specific depletion of Clara cells inhibits both metastasis formation and recruitment of CD11b+ cells [68]. SAA proteins are also chemotactic for other inflammatory cells such as mast cells and T lymphocytes [69, 70], induce the expression of ECM remodeling enzymes [71, 72], and stimulate production of inflammatory cytokines such as TNF-α [68] that promote tumor growth [73, 74]. A number of positively acting feed forward loops exist between TNF-α, SAA, and S100A8/A9 expression, amplifying these inflammatory responses [27]. In addition, CD11b+ Gr-1+ cells also contribute to the expression of pro-inflammatory cytokines in the metastatic niche [19].

### Survival signals

The metastatic niche provides a number of survival functions for DTCs. Integrin-mediated interactions of DTCs with the modified niche ECM induce focal adhesion kinase (FAK) signaling, which promotes both proliferation and survival [75]. The cross-linking of matrix components by lysyl oxidases serves to increase FAK signaling [15]. Matrix stiffness was found to promote chemoresistance in hepatocellular carcinoma cells [76]. Macrophages in the metastatic niche provide survival signals for VCAM-1-expressing tumor cells through direct intercellular interactions [77]. CD11b+ 1 Gr1+ cells are myeloid-derived suppressor cells (MDSC) that inhibit T-cell and NK-cell-mediated immune responses and induce T-cell tolerance in cancer [78–81], thus protecting DTCs from destruction via the immune system.

### Stemness

An idea that has taken root in cancer biology in recent years is that of the cancer stem cell (CSC). Similar to stem cells that both self-renew and give rise to committed progenitors, this hypothesis suggests that populations of tumor cells are organized in a hierarchical manner, with a CSC subpopulation giving rise to the bulk of tumor cells [82]. Importantly, CSCs are operationally distinguished from other tumor cell subpopulations by their ability to initiate the growth of tumors in experimental animals, even as single cells [83]. In contrast, even high numbers of implanted non-CSCs are incapable of establishing tumors. Accordingly, subpopulations of tumor cells defined by expression of defined markers and with tumor-initiating properties *in vivo* have been ascribed stemness properties, including the ability to seed spheroids and expression of genes associated with stem cells [84]. From the point of view of metastasis, the definition of CSCs suggests that DTCs with stemness properties should be the founding cells of secondary tumors [85]. Emerging data suggest that CSC identity is plastic, and that stemness properties need to be maintained and can be acquired by non-CSC populations [3]. Stem cells require a niche to maintain their properties, and components of the metastatic niche have been similarly shown to induce or maintain properties associated with stemness in DTCs [86]. Thus, the metastatic niche may promote the establishment of metastases by endowing DTCs with stemness properties.

The perivascular microenvironment in metastatic niches can initiate and maintain CSC properties. If located in a perivascular location, non-CSC subpopulations can acquire stemness properties [87]. The perivascular niche induced in response to VEGF-A maintains the stemness of skin tumor CSCs [88]. A similar perivascular location maintains glioma CSCs [89] and probably also the CSCs of other tumor types [86].

Hypoxic niches maintain the undifferentiated state of a variety of normal stem cells [90]. Correspondingly, in addition to inducing the formation of niches, hypoxia has been reported to maintain the stemness properties of CSCs [91, 92]. Thus, hypoxia in the metastatic niche would be expected to support metastatic outgrowth by nurturing the metastasis-initiating CSC population, although this remains to be formally demonstrated.

The ECM is a potent regulator of stemness properties. Integrin-mediated activation of FAK signaling in response to ECM microenvironments that typify metastatic niches can enforce CSC identify [93]. Periostin in metastatic niches serves to concentrate and present Wnt ligands, and thereby induces and maintains stem-like metastasis founder cells [7]. Similarly, tenascin-C supports the stemness properties of metastasis founder cells by increasing their responsiveness to Wnt and Notch [6]. Matrix stiffness has been associated with the regulation of CSC properties [76]. Activity of the transcriptional co-activator TAZ is regulated by matrix stiffness [94], and this activity endows breast cancer cells with stemness properties [95].

### Epithelial–mesenchymal transition

Induction of the morphogenetic process of epithelial to mesenchymal transition (EMT) continues to attract much attention as a mechanism that promotes metastasis. During EMT, tumor cells lose epithelial characteristics and acquire mesenchymal properties typified by changes in cellular morphology, altered cell–cell and cell–matrix adhesion, and the development of migratory behavior and invasiveness [96, 97]. Although most studies have focused on the role of EMT in primary tumor invasion, EMT is increasingly being recognized as endowing tumor cells with a number of other properties of relevance to metastasis formation, including the generation and maintenance of CSCs [98–100], escape from signals that induce apoptosis or anoikis such as loss of substrate contact and attack from the immune system [3, 101–103], and resistance against many chemotherapeutic agents [100].

Constituents of the metastatic niche have been associated with the induction of EMT. The remodeling of the ECM that is typical of that found in metastatic niches contributes to EMT [104]. For example, EMT is promoted by the metastatic niche constituent periostin [7]. The lysyl oxidase-like enzyme LOXL2 enhances the activity of the transcriptional repressor Snail1 and thereby induces EMT [105]. MMPs that are activated in the metastatic niche induce EMT [106]. Increased matrix stiffness modifies the response of tumor cells to TGFβ by triggering EMT rather than apoptosis [107]. EMT is strongly and reversibly induced by hypoxia [108]. The presence of inflammatory cytokines in the metastatic niche such as IL-1β can also evoke EMT [109, 110].

It has been proposed that metastases are seeded by CSCs that undergo EMT, allowing then to invade away from the primary tumor and to disseminate [111, 112]. To account for similarities in phenotype between primary tumors and their metastases, as well as the association of EMT with cell cycle arrest (see below), it has been further suggested that the reverse process of mesenchymal to epithelial transition (MET) occurs once they reach the secondary site [111, 112]. To date, there has been little direct experimental evidence to support the induction of MET in DTCs. However, a recent study has shown that the metastatic niche ECM component versican can stimulate MET in tumor cells by reducing phospho-Smad2 levels, resulting in enhanced proliferation and faster metastasis formation [8]. Thus, it is conceivable that the metastatic niche can both stimulate and reverse EMT, possibly depending on its state of maturation. Indeed, it has been suggested that interchangeable EMT and MET partial transitions that are dynamically regulated by the microenvironment determine invasiveness, tumor cell survival, dormancy, and CSC identity in both primary tumors and in metastatic sites [3].

### Dormancy

Niche structures can act to promote DTC survival, but maintain them in a state of dormancy. For example, a perivascular location is required for the survival of DTCs entering the brain, even if they remain dormant [17]. Hypoxia can also induce dormancy [113]. The induction of EMT in DTCs within the niche may also render the cells proliferatively dormant due to increased levels of p16ink4a [114], repression of cyclin D expression [115, 116], and persistent expression of Twist [117]. The presence of cytostatic CD8+ T cells can also induce dormancy [118]. Dormancy confers resistance to cytotoxic chemotherapy on DTCs because their cell cycle arrest or slow proliferative turnover renders DNA-damaging agents that target cycling cells ineffective [119].

On the other hand, dormant DTCs eventually need to be reactivated if they are to grow out as overt metastases. As the metastatic niche matures, a number of niche components can act to release DTCs from dormancy. Alterations in the contingent of cytokines can relieve dormancy induced by CD4+ T cells [120]. Remodeling of the ECM, for example through deposition of collagen type I, has been implicated in releasing tumor cells from dormancy, and acts by enhancing integrin-mediated FAK signaling [121, 122]. The urokinase receptor uPAR activates β1-containing integrins, allowing them to interact with fibronectin, thereby releasing tumor cells from dormancy [123]. Angiogenic dormancy is relieved by the induction of angiogenesis, and the ability of VEGF-A to suppress dormancy [17] partly reflects this.

## Clinical implications

Although basic research into metastatic niches is still at a relatively early stage, it is already clear that the formation and maturation of pre-metastatic and post-dissemination metastatic niches has a number of important potential clinical implications, both from the diagnostic and prognostic perspective, as well as for the design of effective metastasis therapy. By monitoring processes that foster the formation of metastatic niches, it may be possible to identify new and powerful biomarkers that allow metastatic progression to be detected before overt metastasis form. Monitoring the levels of growth factors and cytokines in the blood that induce niche formation is one example. Furthermore, assessment of the levels in the blood of cellular components of metastatic niches such as circulating VEGFR1+ BMDC or CD11b+ myeloid cells may be informative. For the patient, the monitoring of such biomarkers may allow early and more effective therapeutic intervention, before overt metastases are even detected. In addition, these biomarkers may provide a surrogate measure of metastasis formation, facilitating clinical trials of novel therapeutics that target metastatic disease.

Inhibition of stromal progression in metastatic sites as well as the disruption of metastatic niches are possible new approaches to the treatment of metastatic disease. Targeting key immune cells such as CD11b+ myeloid cells may be one option. Interfering with the establishment of an inflammatory milieu through targeting the positive feed forward loops that operate in metastatic niches would be another approach. Once we understand better how dormant DTCs become activated, further therapeutic strategies should become apparent. Therapeutic release from dormancy is likely to sensitize DTCs to chemotherapy. Alternatively, if ways can be identified to therapeutically maintain DTC dormancy indefinitely, cancer could be rendered a chronic rather than a life-threatening disease.

Pre-clinical studies that have targeted pre-metastatic changes in the liver have already proven effective in experiment models. TSU68, a low molecular weight inhibitor of VEGFR-2, PDGFRβ, and FGFR1, reduces CXCL1 expression that is increased pre-metastatically in the liver in response to primary metastatic colon tumors, and suppresses metastasis formation [124]. CXCR2 is the cognate receptor for CXCL1. It is expressed on the colon tumor cells and is functionally required for metastasis formation in this model. TSU68 also reduces the amount of IL-12 in the portal vein and decreases the number of migrating neutrophils in the pre-metastatic liver. Together, these data suggest that TSU68 suppresses pre-metastatic niche formation by inhibiting inflammatory responses induced by the primary tumor [124].

## Conclusions

The concept of the metastatic niche is still discussed controversially, reflecting the still limited number of publications in this area to date, as well as uncertainties regarding, for example, the relevance of DTCs to metastasis formation, the role of BMDC in tumor angiogenesis, and the precise definition of CD11b+ subsets. Nevertheless, this concept and more broadly the notion of stromal progression has turned attention from tumor-intrinsic mechanisms, such as genetic changes that determine metastasis formation, to the importance of microenvironmental regulation of metastasis. Although considerable progress has been made in recent years, there is still much more to learn and a number of key open questions remain to be addressed. Some of the most important of these concern the regulation of DTC dormancy. What microenvironmental changes initiate release from dormancy and outgrowth of metastases, and what external events trigger these microenvironmental changes? To answer these questions, a fuller understanding of stromal progression at secondary sites is required, and how the evolution of metastatic niches is regulated. What are key steps in this evolution, and how does the form and function of the niche change as the niche matures? What defines endogenous niches in different organs, and what constituents do they contribute to the mature niche? What determines where pre-metastatic niches form, and to what degree is their formation relevant to human cancer? What are the minimal requirements for a pre-metastatic niche, and how do DTCs contribute to further maturation of the metastatic niche once they occupy these niches? Answering these questions will not only dramatically enlighten our understanding of the metastatic process but will also provide new strategies for the effective treatment and management of metastatic cancer.
